# A Chrono-Metabolic Approach to Mental Health: Current Perspectives on Circadian Rhythms, Gut Microbiota, and Microbial Metabolites in Mood Disorders

**DOI:** 10.3390/metabo16060400

**Published:** 2026-06-09

**Authors:** Giuseppe Marano, Mariateresa Acanfora, Luca Conci, Gianandrea Traversi, Osvaldo Mazza, Esmeralda Capristo, Eleonora Gaetani, Gianluca Franceschini, Marianna Mazza

**Affiliations:** 1 Department of Neuroscience, Head-Neck and Chest, Section of Psychiatry, Fondazione Policlinico Universitario Agostino Gemelli IRCCS, 00168 Rome, Italy; mariatacanfora@gmail.com (M.A.);; 2 Department of Neuroscience, Section of Psychiatry, Università Cattolica del Sacro Cuore, 00168 Rome, Italy; 3 Unit of Medical Genetics, Department of Laboratory Medicine, Ospedale Isola Tiberina-Gemelli Isola, 00186 Rome, Italy; gianandrea.traversi@gmail.com; 4 Spine Surgery Department, Bambino Gesù Children’s Hospital IRCCS, 00168 Rome, Italy; osvaldo.mazza1973@hotmail.it; 5 Department of Translational Medicine and Surgery, Fondazione Policlinico Universitario A. Gemelli IRCCS, Università Cattolica del Sacro Cuore, 00168 Rome, Italyeleonora.gaetani@unicatt.it (E.G.); 6 Unit of Internal Medicine, Cristo Re Hospital, 00167 Rome, Italy; 7 Breast Surgery Unit, Department of Woman and Child’s Health and Public Health Sciences, Fondazione Policlinico Universitario A. Gemelli IRCCS, 00168 Rome, Italy; 8 Department of Medical and Surgical Sciences, Catholic University of the Sacred Heart, 00168 Rome, Italy

**Keywords:** gut microbiota, major depressive disorder, bipolar disorder, circadian rhythm, chrono-metabolic

## Abstract

**Highlights:**

**What are the main findings?**

- The gut microbiota functions as a rhythmic metabolic organ, whose oscillations interact with circadian biology and influence mood disorder pathophysiology.
- Chrono-disruption alters microbial metabolites, contributing to neuroinflammation, HPA axis dysregulation, and mood instability.

**What are the implications of the main findings?**

- A chrono-metabolic framework supports a shift from static dysbiosis to time-dependent microbiota–host interactions in understanding depression and bipolar disorder.
- Chrono-nutrition, chronobiotics, and metabolomics-driven stratification may enable personalized, precision psychiatry approaches targeting circadian–microbiota alignment.

**Abstract:**

Growing evidence indicates that the gut microbiota is not a static ecosystem but a rhythmic metabolic organ whose oscillatory activity is tightly coordinated with host circadian biology. Disruption of this temporal alignment, through irregular diet, sleep disturbance, shift work, or social jet lag, may profoundly alter microbial composition and the production of neuroactive metabolites. These alterations have emerged as potential contributors to the pathophysiology of mood disorders. This review introduces the concept of chrono-metabolic psychiatry, a framework integrating circadian rhythms, gut microbiota dynamics, and host metabolic signaling in the development and course of depressive and bipolar disorders. In this framework, the term “chrono-metabolic” refers to the integration of biological timing, host metabolic regulation, and microbiota-derived metabolic signaling. Chrono-metabolic psychiatry therefore shifts the focus from static dysbiosis or neurotransmitter imbalance alone to the time-dependent interactions among circadian misalignment, microbial rhythmicity, immune regulation, metabolite production, and affective instability. Diurnal fluctuations in short-chain fatty acids, tryptophan–kynurenine metabolites, bile acids, and microbial-derived neurotransmitters interact with clock gene regulation, hypothalamic–pituitary–adrenal axis activity, neuroinflammation, and synaptic plasticity. Chrono-disruption may represent a transdiagnostic vulnerability factor and may confirm the bidirectional relationship between mood instability and microbiota rhythmicity. Emerging therapeutic implications, including chrono-nutrition, time-restricted feeding, targeted probiotic administration (“chronobiotics”), and the microbiota-modulating effects of psychotropic medications are discussed. By shifting from a compositional to a temporal–metabolic perspective, this model highlights the importance of microbial oscillations rather than static dysbiosis alone. Integrating circadian biology into microbiota research may enable metabolomic stratification and pave the way for precision psychiatry approaches grounded in host–microbe metabolic crosstalk. Future longitudinal and time-resolved multi-omics studies are needed to validate this framework and to translate it into clinically actionable interventions.

## 1. Introduction

The composition of the gut microbiota plays a role in overall health, including mental health [1]. The gut microbiota is essential for host homeostasis: rather than acting as a physical barrier itself, it contributes to the regulation of intestinal epithelial barrier integrity, digestion, nutrient absorption, immune homeostasis, and gut–brain axis signaling [2]. When an imbalance occurs in microbial communities, it is referred to as dysbiosis, which is linked to various mental health issues [3]. The composition of the microbiota is dynamic and influenced by genetics, diet, medications, age, and the environment, and it serves as a hub for neurochemical production: gamma-aminobutyric acid (GABA), produced by *Bifidobacterium* and *Lactobacillus*; serotonin, stimulated by *Lactobacillus* and *Oscillibacter* via tryptophan synthase; and the vagus nerve (VN), influencing not only digestive functions but also memory, emotions, and cognitive processes through the detection of microbial metabolites and interaction with higher brain areas [4]. The microbiota therefore influences mental health through the gut–brain axis, a bidirectional communication system between the gut and the brain: the biochemical pathway of neurotransmitters, the vagus nerve pathway, and the intestinal permeability pathway, with the release of metabolites into the bloodstream [5]. This is why mood disorders (MD) can be viewed as systemic metabolic disorders that are constantly changing. In fact, our body does not function the same way over the course of 24 h, but rather depends on our biological clocks: the master clock, located in the suprachiasmatic nucleus (SCN) of the hypothalamus, which synchronizes the entire body with sunlight; peripheral clocks, since each organ has its own clock; clock genes at the cellular level; and the synchrony between biological clocks and metabolic secretions [6]. Discussing a chrono-metabolic framework allows us to view mental health as a state of well-being and harmony between rhythms (when we perform activities) and metabolism (how the body transforms energy).

The aim of this review is to analyze how mood disorders may be interpreted within a chrono-metabolic framework, in which loss of internal rhythmicity and impaired metabolic efficiency interact with stress biology, immune homeostasis, and microbiota-derived signaling. Circadian disruption may act as a chronic biological stressor, affecting host homeostasis in general and immune regulation in particular. When central and peripheral clocks become misaligned, hypothalamic–pituitary–adrenal axis activity, cortisol rhythmicity, intestinal barrier function, and inflammatory signaling may be altered. This can promote a shift from regulated immune surveillance toward low-grade systemic inflammation, which is increasingly recognized as a relevant contributor to vulnerability, onset, and recurrence in major depressive disorder and bipolar disorder.

For the purposes of this review, chrono-metabolic psychiatry is defined as a conceptual framework that examines how circadian timing, host metabolic regulation, gut microbiota rhythmicity, and microbial-derived metabolites interact in the pathophysiology and treatment of mood disorders. The adjective chrono-metabolic therefore refers to the temporal organization of metabolic processes, including feeding–fasting cycles, peripheral clock activity, microbial oscillations, and metabolite production across the 24 h day. This concept partly overlaps with, but is not identical to, the Circadian Syndrome. The Circadian Syndrome has been proposed mainly as a cardiometabolic construct linking circadian disruption to obesity, hypertension, dyslipidemia, insulin resistance, and sleep disturbance. By contrast, chrono-metabolic psychiatry focuses specifically on mental health outcomes and on the mechanistic interface between circadian biology, the microbiota–gut–brain axis, microbial metabolites, immune regulation, and affective instability. Similarly, while chrononutrition primarily addresses the timing, frequency, and composition of food intake, the chrono-metabolic framework extends this perspective to include microbial rhythmicity, metabolite-mediated signaling, psychotropic drug–microbiota interactions, and metabolomics-based patient stratification [4,5,6].

In this context, circadian rhythmicity refers to the presence of approximately 24 h oscillations in biological, behavioral, microbial, or metabolic processes. Circadian dysregulation refers to the alteration of these rhythms, including phase shifts, amplitude reduction, rhythm flattening, internal desynchronization between central and peripheral clocks, or misalignment between endogenous rhythms and external zeitgebers such as light exposure, sleep timing, and meal timing.

## 2. Materials and Methods

This article is a narrative review supported by a targeted literature search. The aim was to identify and synthesize relevant evidence on the interplay between circadian rhythms, gut microbiota, microbial metabolites, and mood disorders, with particular focus on major depressive disorder and bipolar disorder.

A targeted search was performed in PubMed, Scopus, and Google Scholar. Search terms were used alone and in combination and included: “gut microbiota,” “major depressive disorder,” “bipolar disorder,” “circadian rhythm,” “circadian disruption,” “clock genes,” “microbial metabolites,” “short-chain fatty acids,” “tryptophan,” “kynurenine,” “bile acids,” “chrononutrition,” “time-restricted feeding,” “probiotics,” “chronobiotics,” “metabolomics,” and “precision psychiatry”. The search primarily covered publications from 2010 to 2026. Earlier seminal studies were included when they were considered essential for defining core circadian mechanisms or foundational microbiota–gut–brain axis concepts.

Eligible sources included peer-reviewed original clinical studies, randomized controlled trials, observational studies, systematic reviews, meta-analyses, and mechanistic preclinical or in vitro studies relevant to the conceptual framework of the review. Studies were prioritized when they addressed at least one of the following domains: circadian regulation in mood disorders; gut microbiota rhythmicity; microbial metabolites with potential circadian or neuropsychiatric relevance; diet, sleep, or chrononutrition interventions; probiotic or psychobiotic interventions; metabolomic or multi-omic profiling in depression or bipolar disorder. Exclusion criteria were: non-English articles, conference abstracts without full text, editorials or commentaries without original mechanistic or clinical content, studies unrelated to mood disorders or the microbiota–circadian axis, and articles for which the reported outcomes did not inform the thematic domains of the review.

**Figure 1:**
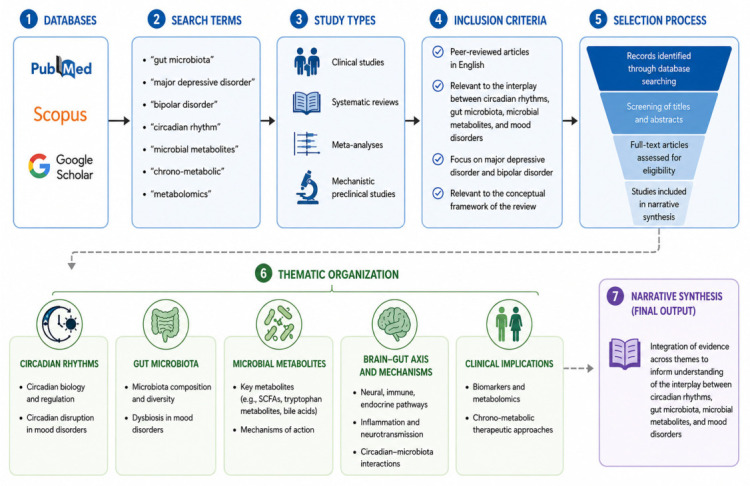
Schematic overview of the targeted literature search and thematic organization of the narrative review. Note. This figure summarizes the targeted search strategy used to identify relevant literature across PubMed, Scopus, and Google Scholar, and the subsequent thematic organization of evidence into the main conceptual domains of the review. It is intended as a narrative synthesis framework and does not represent a formal PRISMA-based systematic review workflow. This figure was generated with the assistance of ChatGPT 5.5/OpenAI under the authors’ guidance. The figure was critically reviewed and edited by the authors, who take full responsibility for its scientific content.

Because this was designed as a narrative review rather than a systematic review, no PRISMA-guided screening, formal risk-of-bias assessment, or quantitative evidence synthesis was performed. The selected literature was organized thematically into mechanistic, translational, and clinical domains. Throughout the manuscript, evidence derived from animal or in vitro studies is explicitly described as preclinical or experimental, whereas findings from human observational or interventional studies are identified as clinical evidence (Figure 1).

## 3. Circadian Rhythms and Mood Disorders

The maintenance of internal temporal order is fundamental for the physiological and psychological health of the organism. Circadian rhythms are endogenous oscillations that coordinate biological processes with the external environment, ensuring that metabolic and behavioral functions occur at the most appropriate time of day. In the context of mental health, the synchronization of these rhythms is essential for emotional stability and cognitive performance. Growing clinical and molecular evidence suggests that the disruption of these biological cycles is not merely a consequence of affective pathologies but a core component of their development. The interaction between core clock genes and neurotransmitter systems creates a bidirectional link where chronobiological stability is intrinsically tied to mood regulation. Therefore, understanding the architecture of the circadian system and its dysregulation is vital for elucidating the pathophysiology of major depressive disorder (MDD) and bipolar disorder (BD).

### 3.1. Organization of the Circadian System

Metabolic, behavioral, and cognitive regulation through the optimization of physiological processes is necessary for adapting to environmental changes. Biological rhythms are controlled by the circadian system, which consists of tissues that exhibit an intrinsic 24 h monitoring activity. The SCN acts as the central pacemaker, coordinating transcriptional–translational feedback loops of core clock genes; within this molecular machinery, CLOCK heterodimerizes with BMAL1 to activate the transcription of Period and Cryptochrome genes through E-box elements [7]. Mutations in CLOCK genes can cause alterations in the period, phase, and amplitude of circadian rhythms [8]. The CLOCK transcription factor or its homolog, the neuronal PAS 2 (NPAS2) protein, forms a heterodimer with the Brain and Muscle Arnt-like Protein 1 (BMAL1) transcription factor to activate the Period (*Per1*, *Per2*, *Per3*) and Cryptochrome (*Cry1*, *Cry2*) genes: Per and Cry are translated into the cytoplasm, where they dimerize, accumulate for approximately 12 h, and return to the nucleus to inhibit their own transcription, forming a negative feedback loop every 24 h [9]. *CLOCK*/*BMAL1* regulate the expression of the nuclear hormone receptors Rev-erbα and Rorα, which respectively activate or inhibit the transcription of BMAL1, creating a feedback loop that stabilizes the core loop [9]. There are also “peripheral clocks,” present in all organs, whose role is to regulate the activity of the organs themselves: CLOCK is responsible for synchronizing the functioning of all peripheral clocks through signals traveling along nerve pathways and through hormone production, enabling the sleep–wake cycle and everything associated with it: changes in heart rate and blood pressure, hormone secretion, regulation of immune function, gastrointestinal activity, and mood [10]. To ensure the optimal functioning of all systems and organs, the SCN must be synchronized with the external environment, keeping time with the environmental clock. These latest preclinical studies emphasize the molecular mechanisms linked to circadian rhythms.

### 3.2. Chronotype

Differences in sleep and activity schedules allow individuals to be classified as “larks”, morning types and “owls”, evening types; this characteristic is known as chronotype, meaning it follows a morning-to-evening circadian continuum [11]. Owls tend to: have a longer circadian rhythm, approximately two hours later than that of morning types; wake up an hour later than larks, with greater difficulty waking up; be exposed to more evening and nighttime light, which amplifies the delay in the sleep window; have a stronger homeostatic drive (the pressure to sleep while awake) in larks, which dissipates more quickly, compared to evening types, who delay going to bed [12]. Chronotype is 50% heritable: approximately 350 associated genes have been identified, including *Per2*, *Per3*, *Cry1*, and variants related to retinal ganglion cells and the metabolism of nicotine and caffeine; Lark-type chronotypes peak around age 20 and then shift toward morning-type chronotypes in old age; men tend to have later chronotypes than women until age 40, when the difference disappears [13]. The discrepancy between the biological clock and social obligations (school or work) is called social jet lag and is more pronounced in evening types: night owls accumulate sleep debt during the workweek, attempting to catch up on their days off, and consume more caffeine, which reduces the homeostatic drive, lengthening the circadian period and delaying the sleep phase [14].

### 3.3. Circadian Dysregulation in Major Depressive Disorder and Bipolar Disorder

Circadian rhythms play a crucial role in the etiology and treatment of mood disorders as highlighted by the clinical trials to be cited below. There is a biological bidirectional relationship, both from the clock to mood, since sleep disturbances and diurnal variations are symptoms of MDD and BD and, in healthy individuals, the biological clock regulates mood and motivation; and from mood to the clock, since acute or repeated stress can alter circadian rhythms [15]. Certain genetic polymorphisms of BMAL1 and Npas2 have been associated with MDD, and others of Clock with BD: these genes are expressed primarily in brain tissue, for example, Npas2 in the nucleus accumbens (NAc), a key region in reward processing [16]. MDD is characterized by somatic symptoms: sleep disturbances, with insomnia in 40% of cases and a slowed circadian rhythm, worsening of symptoms in the morning or evening, changes in appetite, changes in social interactions, and alterations in blood pressure, body temperature, or hormone levels [17]. These elements indicate central rhythmic dysregulation, suggesting that circadian disruption is a factor underlying the development and maintenance of MDD [18]. Dysregulation of biological rhythms is also common in patients with BD, regardless of mood state and medications taken. Owls are associated with a poorer prognosis in patients with BD, with the following characteristics: biological alterations, namely a smaller pineal gland volume and lower nocturnal melatonin production; specific symptoms, namely higher levels of anxiety, reduced overall functioning, and more episodes of mood swings; physiological alterations, namely inflammatory processes that mediate the relationship between chronotype and symptom severity, and a higher BMI consistent with the general population [19,20,21,22].

## 4. Circadian Rhythmicity of Gut Microbiota

The biological clock and the gut microbiota are interconnected through reciprocal host–microbe signaling. In humans and animal models, the gut microbiota exhibits daily rhythms in both taxonomic abundance and metabolic function, with feeding–fasting cycles acting as major entraining signals [23]. Diets high in fat or sugar can blunt microbial oscillations and alter host circadian metabolic function, whereas structured feeding–fasting patterns, including intermittent fasting, may partially restore rhythmic microbial activity [23,24]. Dietary composition is therefore a major determinant of microbiota structure and function in humans and may also influence sleep-related outcomes. In particular, dietary patterns rich in prebiotics, fibers, and polyphenols, such as the Mediterranean diet, may promote SCFA-producing taxa and support the production of microbial-derived metabolites, including short-chain fatty acids and GABA-related pathways, which are potentially relevant to sleep regulation [25].

Beyond diet, experimental evidence indicates that circadian dysregulation itself can alter microbiota rhythmicity and intestinal barrier function [26]. In animal models, circadian disruption and clock-gene perturbation have been associated with impaired epithelial barrier regulation, increased intestinal permeability, altered microbial composition, and inflammatory phenotypes [27]. Therefore, claims regarding *Per*/*BMAL1* disruption, intestinal hyperpermeability, and systemic inflammation should be interpreted primarily as preclinical mechanistic evidence rather than direct clinical proof in patients with mood disorders. Importantly, systemic inflammation should not be interpreted only as a downstream consequence of mood disorders. In vulnerable individuals, inflammatory activation may precede the onset or recurrence of major depressive disorder and bipolar disorder, acting as a permissive biological condition that interacts with circadian misalignment, stress exposure, and microbiota-related barrier dysfunction [24].

Microbial metabolites may also participate in host circadian regulation; for example, administration of short-chain fatty acids, such as butyrate, has been shown in experimental models to induce phase shifts in peripheral clocks and to increase NREM sleep in rats [25]. Bidirectional effects also exist: in animal models, sleep fragmentation alters gut microbial composition, including the Firmicutes/Bacteroidetes ratio [28].

The circadian rhythmicity of the gut microbiota extends beyond compositional fluctuations and includes coordinated changes in metabolic activity, bacterial load, intestinal barrier function, and host–microbe signaling across the 24 h cycle. Disruption of these temporal patterns through irregular eating habits, sleep deprivation, or circadian misalignment may therefore contribute to loss of microbial rhythmicity, impaired barrier integrity, and systemic inflammation, although the direct relevance of these mechanisms to mood disorders requires further longitudinal clinical validation.

Table 1 summarizes the key features of gut microbiota circadian dynamics, underlying mechanisms, and their implications for host metabolic and mental health.

## 5. Microbial Metabolites as Chrono-Metabolic Signals

**Figure 2:**
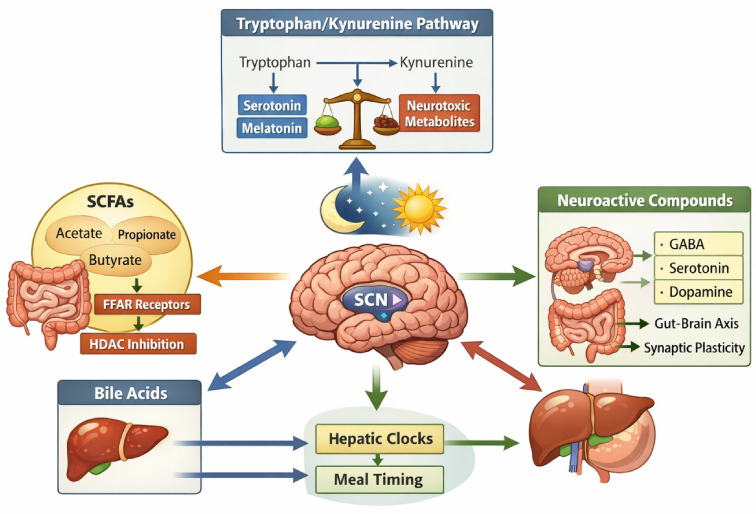
Integrated model of microbial metabolites as core mediators of chrono-metabolic signaling. Note. The diagram highlights four key pathways: SCFAs (acetate, propionate, butyrate) regulate satiety hormones and clock genes (*Per*, *BMAL1*) via FFAR receptors and HDAC inhibition. Tryptophan/Kynurenine pathways balance serotonin/melatonin synthesis against neurotoxic metabolites. Bile Acids synchronize hepatic clocks with meal timing and microbial activity. Neuroactive Compounds (GABA, Serotonin, Dopamine) modulate the gut–brain axis and synaptic plasticity in coordination with the central clock (SCN). This figure was generated with the assistance of ChatGPT/OpenAI under the authors’ guidance. The figure was critically reviewed and edited by the authors, who take full responsibility for its scientific content.

The gut microbiota functions as a rhythmic endocrine organ, generating a diverse array of molecules that sync peripheral oscillators with environmental cues, primarily meal timing. These microbial metabolites serve as critical trans-genomic signals, biochemically preparing the host for energy intake and bridging the gut–brain axis. The integrated pathways through which key microbial metabolites (including SCFAs, tryptophan derivatives, and bile acids) influence central and peripheral circadian regulation, neurotransmitter rhythms, and metabolic homeostasis are visually synthesized in the comprehensive mechanistic model presented in Figure 2.

### 5.1. SCFAs

The gut microbiota, particularly *Faecalibacterium*, *Bacteroides*, *Roseburia*, *Phascolarctobacterium*, and *Parabacteroides*, produces SCFAs, including acetate, propionate, and butyrate, which are derived from the fermentation of indigestible dietary fiber in the colon [29]. SCFAs bind to specific G-protein-coupled receptors, particularly free fatty acid receptors 2 and 3 (FFAR 2/3): the binding of SCFAs to receptors on colonic L cells stimulates the release of satiety hormones such as peptide YY (PYY), which reduces energy intake by acting on the hypothalamus, and glucagon-like peptide-1 (GLP-1), which acts as an incretin, stimulating insulin production; propionate, in particular, binds to the FFA3 receptor in adipocytes, stimulating the production of leptin, the hormone that signals satiety in the arcuate nucleus of the hypothalamus [29]. SCFAs, synthesized in the colon, can reach the brain via specific transporters in intestinal and endothelial cells that facilitate their absorption and passage through the blood–brain barrier (BBB); once across the BBB, they contribute to its integrity and influence neuronal activity, by promoting synaptic plasticity [30]. Dos Santos and Vasylyshyn reviewed studies on SCFAs and circadian rhythms in mice: animal studies indicate that increased SCFA levels through supplementation correlate with a phase advance of circadian rhythms in various tissues, including the liver, kidneys, and colon; via FFAR2/3 receptors, SCFAs activate the cAMP Response Element-Binding protein (CREB), which binds to the promoters of the clock genes *Per1* and *Per2*; in the intestine, SCFAs act as histone deacetylase (HDAC) inhibitors, increasing histone acetylation and improving chromatin accessibility of the *BMAL1*, *Per1*, and *Cry1* genes; SCFA-induced changes in colonic pH reset peripheral oscillators; by stimulating the release of GLP-1 and insulin, SCFAs synchronize the hepatic clock via Per modulation; SCFAs do not appear to directly entrain the SCN, which responds primarily to light, although they may modulate peripheral clocks and systemic metabolic signaling [31,32,33]. In a double-blind study involving oral butyrate supplementation, Firoozi et al. detected a significant upregulation of the core clock genes *Cry1*, *Cry2*, *Per1*, and *BMAL1* [34]. Most evidence linking SCFAs to peripheral clock entrainment and HDAC-related regulation derives from animal and experimental studies, while direct evidence in psychiatric populations remains limited.

### 5.2. Tryptophan and Kynurenine Pathways

Tryptophan is the primary precursor of serotonin and melatonin: serotonin governs the waking phase, and its conversion to melatonin is the key signal for the onset of the evening rest phase; the availability of tryptophan in the brain across the blood–brain barrier depends on competition with other amino acids and on the intake of carbohydrates and proteins during the day [35]. In the presence of stress or inflammation, tryptophan is diverted from serotonin synthesis toward the kynurenine pathway: the imbalance favoring quinolinic acid, which is neurotoxic, at the expense of kynurenic acid, which is neuroprotective, acts as a metabolic alarm signal that can desynchronize circadian rhythms; activation of the enzyme indoleamine 2,3-dioxygenase (IDO) reduces the amount of tryptophan available for melatonin, contributing to sleep disturbances; the gut microbiota regulates tryptophan levels through absorption or diversion toward the kynurenine pathway; fasting or calorie-restricted diets influence the IDO enzyme, suggesting that meal timing can be used to influence tryptophan metabolism and circadian signals, acting as a chronobiological signal [35,36].

### 5.3. Bile Acids and Internal Clock Crosstalk

Bile acids act as trans-genomic molecules, as they biochemically prepare the host for energy intake. Their effectiveness depends strictly on the synchronization between the environment, eating behavior, and sleep. Bello et al. observed that the rhythmicity of bile acids in male humans is drastically reduced in the absence of external signals, confirming that their presence in the blood is under environmental control (meals or light–dark cycle) [37]. Despite the consumption of three daily meals, only one daily peak was observed for all bile acids: conjugated bile acids begin to rise before lunch and peak after dinner, reflecting absorption in the small intestine; unconjugated bile acids, produced by the bacterial enzyme bile salt hydrolase (BSH) in the colon, peak between nighttime and late morning, suggesting peak microbiota activity during the night [37]. Although circulating bile acid levels are strongly shaped by environmental cues such as meal timing and light–dark cycles, hepatic bile acid synthesis and enterohepatic transport are also regulated by circadian nuclear receptors, including Rev-ERBα and RORα. In this context, synthesis refers primarily to the hepatic production of bile acids through enzymes such as CYP7A1 and CYP8B1, whereas transport refers to the coordinated activity of hepatic and intestinal bile acid transport systems involved in enterohepatic circulation. Rev-ERBα can repress BMAL1 and modulate *CYP7A1* expression, thereby limiting bile acid accumulation, while RORα contributes to the regulation of *CYP8B1* and cholic acid synthesis [38]. Bile acids themselves contribute to these patterns through a negative feedback loop by inhibiting CYP7A1 [38]. The loss of coordination between the CLOCK circadian clock and bile acids could influence the risk of developing metabolic diseases, obesity, and dyslipidemia observed in shift workers and those suffering from chronic sleep deprivation [39].

### 5.4. Microbial-Derived Neuroactive Compounds

The gut microbiota acts as both a producer and a modulator of neuroactive compounds that influence mood: GABA, converted from glutamate by strains such as *Lactobacillus* and *Bifidobacterium*, is the primary inhibitor of the nervous system, with which it communicates via the vagus nerve; 95% of serotonin is produced by the gut, which regulates tryptophan availability and serotonin turnover via the Serotonin Transporter (SERT) and the enzymes Monoamine Oxidase (MAO) and IDO, which increase in cases of intestinal dysbiosis, thereby impairing serotonin production. Some Bacillus and Serratia species directly synthesize dopamine (DA) or process L-DOPA, whose levels fluctuate according to microbial circadian rhythms; in the brain, serotonin, DA, and norepinephrine exhibit release rhythms coordinated by the SCN [40].

## 6. Chrono-Metabolic Pathways Linking Microbiota and Mood

The HPA axis is the primary pathway through which stress is regulated. The gut microbiota influences the HPA axis: in conditions of dysbiosis, the HPA axis becomes hyperactive, leading to chronic cortisol secretion, which under normal conditions follows a circadian rhythm peaking upon waking; flattening this rhythm leads to chronic fatigue and reduced emotional resilience, affecting mood [3]. Chronic stress, aging, and nutrition can cause DNA methylation, “silencing” clock genes; in particular, *BMAL1* methylation differs significantly in patients with bipolar disorder compared to controls [28]. There is also a negative correlation between the bacterial diversity of the gut microbiota and *BMAL1* methylation: metabolites from gut bacteria and processed nutrients, such as polyphenols, can influence gene regulation, affecting brain health and sleep [27]. In cases of dysbiosis, a compromised intestinal barrier (leaky gut), or disrupted circadian rhythms, there may be an increase in pro-inflammatory cytokines and their entry into the bloodstream, such as lipopolysaccharides (LPS): LPS cross the blood–brain barrier (BBB) and activate microglia, the immune cells of the central nervous system (CNS). This chronic neuroinflammation alters neurotransmitter (NT) synthesis; for example, by diverting tryptophan toward the neurotoxic kynurenine pathway rather than toward serotonin, thereby influencing mood states [30,31]. This inflammatory activation may precede, rather than simply follow, the clinical expression of mood disorders. In particular, low-grade systemic inflammation has been proposed as a vulnerability factor for both major depressive disorder and bipolar disorder, potentially amplifying stress sensitivity, HPA axis dysregulation, neuroinflammatory signaling, and alterations in tryptophan–kynurenine metabolism [24].

Speaking of metabolic peptides, in preclinical research, butyrate improves cognitive performance by promoting histone acetylation and stimulating synaptic plasticity, particularly the expression of Brain-Derived Neurotrophic Factor (*BDNF*), which, in turn, follows a circadian rhythm [41]. In fact, the pathways of neuronal plasticity (particularly neurotrophin production, of which *BDNF* is the most studied) and the apoptosis pathway are also under the control of *CLOCK*/*BMAL1* [42]. According to Marrocco et al., acetate shows potential in reducing neuroinflammation and improving synaptic function, and an increase in secondary bile acids, caused by an abundance of gut bacteria, can compromise the integrity of the blood–brain barrier [41]. In animal models with *CLOCK* mutations, peptides such as CCK, orexin, and ghrelin become dysrhythmic: in particular, CCK is regulated by CLOCK, and its reduction is linked to manic states [43]. The molecular clock influences mood by directly affecting the degradation of neurotransmitters: the *MAOA* gene, in fact, is a direct target of *BMAL1* and *PER2* [40]. There is also a hypothesis suggesting that mitochondrial dysfunction is present in psychiatric disorders [44]: in this regard, it should be noted that the circadian cycle is coupled to cellular redox status via *CLOCK*/*BMAL1* [45]. Evidence linking butyrate-related histone acetylation, *BDNF* modulation, and chrono-metabolic signaling to mood disorders is currently largely preclinical and should therefore be interpreted as mechanistic support rather than direct clinical proof.

## 7. Chrono-Disruption as a Transdiagnostic Risk Factor

Daily routines may cause the biological clock to become desynchronized from the environmental clock, with significant consequences for well-being and health. An increasing number of individuals are exposed to night work, when the endogenous biological clock promotes sleep. Similarly, young people frequently engage in social activities at night, and long-distance flights across multiple time zones create a mismatch between internal biological time and the external time of the destination country [13]. Additional lifestyle and environmental stressors, such as prolonged exposure to blue artificial light and chronic stress, may further disrupt natural circadian rhythms [24]. Irregular eating patterns are also relevant: consuming meals at times that are misaligned with metabolic rhythms, particularly at night, may disrupt peripheral clocks in the digestive system and create internal desynchronization between the brain’s master clock and peripheral metabolic oscillators [28]. Scientific evidence increasingly indicates that circadian disruption is implicated in several health conditions, including sleep disorders, cardiovascular disease, infections, metabolic syndrome, and neuropsychiatric disorders such as depression [7,10,18].

**Figure 3:**
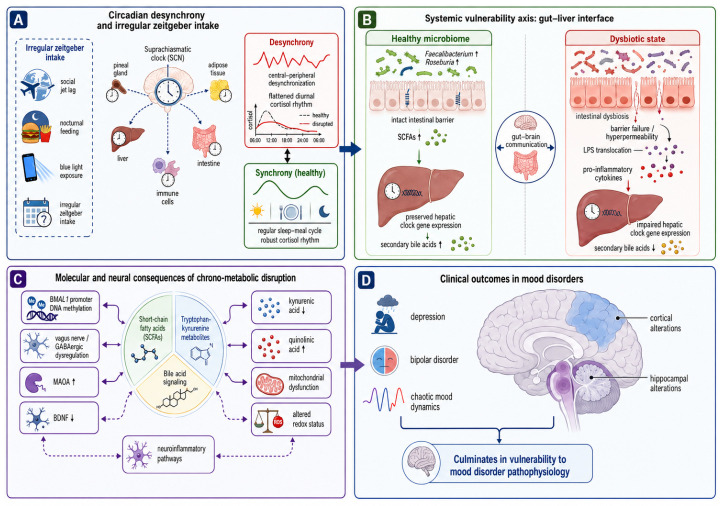
Integrated chrono-metabolic framework for mood disorders. Note. Schematic overview of the bidirectional communication within the microbiota–gut–brain axis under conditions of circadian synchrony and disruption. Panel (**A**) illustrates pathophysiological drivers such as social jet lag, nocturnal feeding, and blue light exposure which precipitate central peripheral desynchronization and the subsequent flattening of the diurnal cortisol rhythm. Panel (**B**) delineates the systemic vulnerability axis where a healthy microbiome characterized by high *Faecalibacterium* and *Roseburia* abundance maintains intestinal barrier integrity and high short chain fatty acid levels. In contrast, microbial dysbiosis promotes intestinal hyperpermeability and the systemic translocation of lipopolysaccharides and pro-inflammatory cytokines, leading to compromised hepatic clock gene expression and reduced secondary bile acid synthesis. Panel (**C**) highlights the molecular consequences of chrono metabolic disruption, including aberrant DNA methylation of the *BMAL1* promoter and GABAergic dysregulation via vagus nerve communication. At the synaptic level, the shift from neuroprotective kynurenic acid toward neurotoxic kynurenine metabolites such as quinolinic acid is shown alongside increased *MAOA* activity and decreased *BDNF* expression. These pathways, coupled with mitochondrial dysfunction and altered cellular redox status, culminate in the clinical outcomes presented in Panel (**D**), characterized by chaotic mood dynamics and structural alterations in cortical and hippocampal regions associated with depression and bipolar disorder. This figure was generated with the assistance of ChatGPT/OpenAI under the authors’ guidance. The figure was critically reviewed and edited by the authors, who take full responsibility for its scientific content.

The complex interplay between circadian rhythms, gut microbiota, and mood regulation can be conceptualized as a multidimensional network in which clock genes, microbial oscillations, intestinal barrier function, immune signaling, and metabolic pathways are tightly interconnected. As summarized in Figure 3, social jet lag, nocturnal feeding, sleep disruption, blue-light exposure, and chronic stress may desynchronize central and peripheral clocks, flatten cortisol rhythmicity, and disrupt microbial oscillations [13,24,28]. These alterations may promote intestinal hyperpermeability, lipopolysaccharide translocation, systemic inflammation, and neuroinflammatory signaling [24,30,31]. At the molecular level, microbial metabolites such as short-chain fatty acids, tryptophan-kynurenine derivatives, bile acids, and microbial-derived neuroactive compounds may influence circadian regulation, immune activation, neurotransmitter availability, mitochondrial function, and synaptic plasticity [29,30,31,32,33,34,35,40]. Additional preclinical evidence links clock-gene disruption to altered neuropeptide signaling, BDNF-related plasticity, monoaminergic metabolism, and cellular redox regulation [41,42,43,44,45]. This framework supports chrono-disruption as a transdiagnostic vulnerability factor, while also emphasizing that several of these mechanistic links remain primarily supported by preclinical or translational evidence and require validation in longitudinal clinical studies.

## 8. Therapeutic Implications

The integration of chronobiology and microbiology into clinical practice offers a transformative perspective on the management of mood disorders. Given the bidirectional nature of the microbiota–gut–brain axis and its profound regulation by the circadian system, therapeutic strategies are increasingly moving toward interventions that simultaneously address metabolic and rhythmic dysfunctions. Traditionally, psychiatric treatments have focused on neurochemical stabilization; however, the emerging evidence presented in this framework suggests that restoring circadian synchrony and microbial homeostasis may significantly enhance treatment efficacy. This chapter explores the potential of chrononutrition, targeted chronobiotics, and the role of psychotropic medications in modulating the microbial landscape. By bridging the gap between molecular biology and clinical intervention, these approaches aim to establish a more holistic and personalized model of psychiatric care that accounts for the rhythmic and metabolic individuality of the patient.

### 8.1. Chrono Nutrition and Time Restricted Feeding

Recent research is analyzing how restriction interventions on the caloric intake window, without reducing total energy intake, improve metabolic and circadian parameters in animal models with obesity and chronodisruption. In obese mice, an 8 h feeding window during the early phase of activity (early time-restricted feeding, or TRF) has been shown to reduce body weight, inflammation, insulin resistance, and realign hepatic circadian rhythms compared to ad libitum feeding or late TRF [46]. Similarly, in Drosophila with high-fat diet-induced metabolic damage, a 12 h TRF may attenuate triglyceride levels and hyperglycemia and partially restore the amplitude of peripheral clock gene rhythms [47]. However, some studies illustrate how TRF during murine early life produced opposite effects, with persistent metabolic disturbances and an “unfavorable” microbiota profile in adulthood [48,49]. These findings suggest that meal timing may represent a modifiable factor influencing circadian and metabolic homeostasis, although its clinical applicability in psychiatric populations remains to be established.

### 8.2. Targeted Chronobiotics and Probiotics

Evidence supports the emerging concept of chronobiotic probiotics, defined here as probiotic interventions that may influence both gut microbial ecology and circadian-related host pathways. In this context, the term “chronobiotic” does not simply refer to a probiotic with general psychobiotic properties, but to a microbial intervention with potential effects on circadian rhythmicity, clock-gene expression, sleep-related physiology, or metabolite-mediated host–microbe signaling. The PROVIT-CLOCK trial showed that a multispecies probiotic add-on intervention in patients with major depressive disorder was associated with modulation of clock gene expression and correlations between fecal or serum metabolites and circadian gene expression, supporting a possible metabolite-mediated mechanism [50]. However, the available human evidence does not yet demonstrate that individual probiotic strains directly regulate *CLOCK* or *BMAL1* expression in isolation. Therefore, these findings should be interpreted as formulation-level evidence rather than definitive proof of strain-specific *CLOCK*/*BMAL1* regulation.

Consistent clinical and preclinical evidence suggests that probiotic interventions may also influence outcomes closely related to chrono-metabolic regulation, including depressive symptoms, fatigue, sleep continuity, stress-related biomarkers, inflammatory signaling, and tryptophan–serotonin metabolism [51,52,53,54]. Notably, experimental animal studies further suggest that clock-gene-targeted probiotic strategies may exert melatonin-like effects on circadian rhythmicity and cognitive performance [55]. Although these findings have not yet been fully translated into psychiatric clinical populations, they strengthen the biological plausibility of probiotics as potential chrono-metabolic modulators.

To improve clarity and distinguish clinical from preclinical evidence, Table 2 summarizes the probiotic strains or formulations currently discussed in relation to circadian–microbiota–mood interactions. The table reports the strain or formulation, study design, population or experimental model, and main chrono-metabolic or mood-related effects. This evidence map highlights that human data remain limited and largely formulation-based, whereas several mechanistic effects on circadian rhythmicity, inflammation, and microbial oscillations are still supported mainly by preclinical studies.

### 8.3. Microbiota-Modulating Effects of Psychotropic Drugs

The possible links between psychotropic assumption and specific microbial signatures has already been a subject of study in the past years. Recent studies have shown that treatment with atypical antipsychotics is particularly associated with dysbiosis characterized by reduced richness (particularly in women) and alterations in taxa such as Lachnospiraceae, Akkermansia, and Sutterella [56], while also reducing alpha diversity in a dose-dependent manner [57]. Microbial and metabolomic profiles can also distinguish responders from non-responders to SSRIs, indicating a predictive role for personalized treatment strategies [58].

### 8.4. Toward Personalized Chronometabolic Interventions

**Figure 4:**
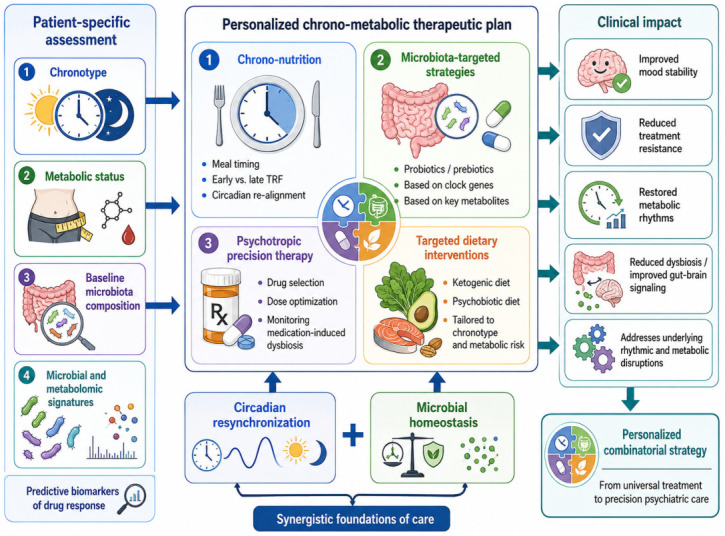
Chrono-metabolic therapeutic interventions. Note. The model illustrates a multi-target approach combining chrono-nutrition to realign metabolic rhythms, targeted chronobiotics to modulate clock genes and mood, and psychotropic drug monitoring to manage medication-induced dysbiosis. These elements converge into personalized interventions that integrate patient-specific chronotypes and microbial signatures into a systemic model of psychiatric care. AI disclosure: This figure was generated with the assistance of ChatGPT/OpenAI under the authors’ guidance. The figure was critically reviewed and edited by the authors, who take full responsibility for its scientific content.

Evidence converges on a model in which chronotype, metabolic status, microbiota composition, and pharmacological response may be incorporated in a single therapeutic plan. Considering the growing ability to use microbial and metabolomic signatures as predictive biomarkers of drug response [58,59,60,61], personalized combinatorial strategies may be viable in the future. This would include meal timing (early vs. late TRF), probiotics/prebiotics selected based on clock genes or key metabolites, selection and dosing of psychotropic drugs according to microbial profile and targeted dietary interventions (e.g., ketogenic or “psychobiotic” diets) tailored to chronotype and metabolic risk profile. The convergence of these diverse therapeutic avenues spanning from the temporal regulation of nutrient intake to the precision modulation of the microbial landscape establishes a new frontier in psychiatric care. As synthesized in Figure 4, this chronometabolic framework shifts the clinical focus toward a multitarget approach where circadian resynchronization and microbial homeostasis act as synergistic pillars. By integrating patient-specific data including chronotype, metabolic signatures, and baseline microbiota composition, clinicians can transition from a universal model to personalized combinatorial strategies. This approach not only addresses the symptomatic manifestations of mood disorders but also targets the underlying rhythmic and metabolic disruptions that drive disease progression and treatment resistance.

## 9. Metabolomics and Precision Psychiatry

The transition toward precision psychiatry necessitates the integration of complex biological data to overcome the limitations of traditional symptom-based diagnostic frameworks. Metabolomics serves as a critical bridge in this process, offering a high resolution snapshot of the functional interactions between the host genome, the gut microbiome, and environmental zeitgebers. By quantifying the end products of cellular processes, metabolomic profiling captures the dynamic state of the organism, reflecting both the genetic predisposition and the real time physiological response to circadian disruption. This chapter delineates the strategic role of metabolomics and multi-omics integration in patient stratification and the discovery of robust biomarkers that account for the temporal and metabolic heterogeneity of mood disorders.

### 9.1. Feasible Roles of Metabolomics and Multi-Omics Integration

Psychiatric disorders are characterized by marked clinical and biological heterogeneity, with MDD representing one of the most paradigmatic examples due to its highly variable clinical presentation. This heterogeneity limits the effectiveness of standard treatments and highlights the need to move beyond purely symptom-based classifications. In this context, precision psychiatry aims to incorporate multi-omics frameworks that combine genomics, epigenomics, proteomics, metabolomics, and microbiomics into patient stratification through the identification of reliable biomarkers [62]. Among -omics technologies, metabolomics appears particularly promising, as it provides a dynamic readout of the organism’s functional state. Within a multi-omic framework, this approach enables the identification of biologically meaningful patient profiles, supporting more accurate diagnosis and personalized treatment strategies. Notably, recent evidence indicates that metabolomic profiling can stratify MDD into distinct biological subtypes characterized by specific metabolic alterations, supporting the feasibility of biomarker-driven classification and precision psychiatry approaches [63,64]. To clarify the potential clinical relevance of metabolomic profiling for patient stratification, Table 3 summarizes the main metabolite classes implicated in biologically distinct depressive phenotypes. The table highlights how specific metabolic alterations may reflect inflammation-related, bioenergetic, lipid-related, microbiota-dependent, circadian, or oxidative-stress-related subtypes of major depressive disorder.

### 9.2. Biomarker Discovery

The identification of reliable biomarkers represents a critical step toward translating chrono-metabolic psychiatry into clinical practice. In recent years, metabolomics has emerged as a particularly promising tool for biomarker discovery in mood disorders, as it captures the dynamic biochemical output of the interaction between host metabolism, circadian regulation, and gut microbiota activity. Unlike static genomic or microbiomic profiles, metabolomic signatures provide a time-sensitive snapshot of physiological states, making them especially suitable for investigating chrono-biological processes.

A growing body of evidence indicates that distinct metabolomic patterns can differentiate MDD from bipolar disorder BD, as well as identify subtypes within these conditions. Panels based on circulating metabolites, such as amino acids, lipids, bile acids, and SCFAs have demonstrated clinically relevant diagnostic accuracy in distinguishing psychiatric phenotypes. Alterations in tryptophan metabolism, including shifts toward the kynurenine pathway, have been consistently associated with depressive states, reflecting the interplay between inflammation, circadian disruption, and neurotransmitter availability.

Emerging studies suggest that metabolomic profiles may also predict treatment response. For example, baseline microbial and metabolic signatures have been associated with differential response to SSRIs, indicating that the gut microbiota–metabolite axis may influence pharmacodynamics and therapeutic efficacy. This opens the possibility of using metabolomic biomarkers not only for diagnostic stratification but also for guiding personalized treatment strategies [65,66].

Within a chrono-metabolic framework, the temporal dimension of biomarker assessment becomes crucial. Many candidate biomarkers, including SCFAs, bile acids, and hormonal mediators, exhibit diurnal oscillations that may be flattened or phase-shifted in mood disorders. Consequently, time-of-day-controlled sampling is essential to avoid misinterpretation of results and to fully capture biologically meaningful variations. Integrating longitudinal and time-resolved metabolomic data may further enhance the identification of stable yet dynamic biomarkers that reflect both trait and state components of psychiatric illness. The integration of metabolomics with other omics technologies, such as genomics, epigenomics, and microbiomics, within multi-omics frameworks holds promise for identifying composite biomarker signatures with higher sensitivity and specificity. Such approaches may enable the stratification of patients into biologically defined subgroups, paving the way for precision psychiatry models that incorporate circadian phenotypes, microbial profiles, and metabolic states into individualized care pathways [67].

Despite these advances, several challenges remain, including the need for standardization of analytical platforms, validation in large and diverse cohorts, and the development of clinically accessible assays. Addressing these limitations will be essential to move from exploratory biomarker discovery to routine clinical implementation.

To provide an integrated overview of the evidence discussed throughout this review, Table 4 summarizes the principal clinical, translational, and preclinical findings linking circadian rhythms, gut microbiota, microbial metabolites, and mood disorders. The table highlights the current level of evidence supporting each mechanistic domain and distinguishes findings derived from human studies from those primarily supported by experimental models.

## 10. Limitations and Challenges

Despite progress in understanding the microbiota–circadian rhythm axis, a number of methodological challenges are slowing down the clinical translation of these findings. One of the main issues is the high heterogeneity of microbiome studies, most of which are conducted in laboratory animal models, and thus often result from differences in sequencing techniques, uncontrolled dietary variables and host genetic factors that complicate the identification of universal biomarkers. The current literature lacks longitudinal experimental designs, making it difficult to determine whether dysbiosis and circadian misalignment are primary causes or consequences of mood disorders. It is also essential to implement time-of-day controlled sampling protocols, as both microbial composition and metabolites such as SCFAs and bile acids fluctuate rhythmically over a 24 h period. Sampling carried out at incorrect times may produce altered results, masking the true dynamic nature of these biological systems.

## 11. Conclusions: Future Perspectives in Chrono-Metabolic Psychiatry

The integration of chronobiology and research into the gut–brain axis represents a paradigm shift towards a more holistic and precise form of chrono-metabolic psychiatry. Mental health is certainly a product of brain neurochemistry, but it also depends on the synchronization of all our body’s biological clocks. Therefore, the future of precision psychiatry lies in the development of personalized treatment plans: from TRF to the use of ‘chronobiotics’ and specific probiotics, right through to the calibration of psychotropic drugs based on the patient’s metabolic and microbial profile.

Addressing chronodisruption as a transdiagnostic risk factor will enable us to move beyond symptom management, aiming instead to restore biological harmony and improve long-term emotional resilience. The transition from a universal diagnostic model toward a precision framework requires the seamless integration of temporal, microbial, and metabolic data.

## Figures and Tables

**Table 1:** Circadian dynamics of the gut microbiota.

Feature	Mechanism	Effect on Health
Compositional Variation	Bacterial populations oscillate over 24 h.	Maintains metabolic homeostasis and the proper degradation of nutrients.
Total Bacterial Load	Bacterial density peaks during the host’s active phase and decreases during the rest phase.	Coordinates energy extraction from food with the host’s energy expenditure.
Clock Genes	*BMAL1* and *Per1*/*2* in the gut drive local rhythms	If clock genes are mutated or absent, bacterial oscillations disappear, causing dysbiosis.
Feeding–Fasting Cycle	Nighttime fasting allows for the proliferation of species that degrade intestinal mucus, protecting the barrier.	Irregular feeding flattens oscillations, promoting inflammation.
SCFAs	The production of SCFAs follows a daily rhythm based on the fermentation of fibers.	SCFAs send signals to peripheral tissues to synchronize their internal clocks.
Intestinal Barrier	Intestinal permeability is rhythmic and regulated by melatonin and clock genes.	Sleep deprivation disrupts this rhythm, leading to “leaky gut” and systemic inflammation.

Note. From the rhythmic variation of bacterial populations to the expression of local clock genes such as *BMAL1*, these oscillations ensure that metabolic energy extraction is aligned with the host’s activity. When these rhythms are disrupted, whether due to irregular eating habits or sleep deprivation, the resulting dysbiosis and increased intestinal permeability can trigger systemic inflammation and metabolic imbalances. Abbreviations. SCFAs: Short-chain fatty acids; *BMAL1*: Brain and Muscle ARNT-Like 1 (core clock gene); *Per1*/*2*: Period circadian regulator genes 1 and 2; Cry1: Cryptochrome circadian regulator 1.

**Table 2:** Chronobiotic and psychobiotic strains/formulations relevant to circadian–microbiota–mood research.

Strain/Formulation	Study Design	Population/Model	Reported Chrono-Metabolic or Mood-Related Effects	Evidence Level
Multispecies probiotic formulation used in the PROVIT-CLOCK trial	Randomized, double-blind, placebo-controlled add-on trial	Patients with major depressive disorder	Modulation of clock gene expression; correlations between fecal or serum metabolites and circadian gene expression	Human clinical; formulation-level evidence [50]
Probiotic intervention associated with depressive symptoms, fatigue, and sleep continuity	Clinical interventional study	Individuals with depressive or sleep-related symptoms	Improvements in depressive symptoms, fatigue, and sleep continuity	Human clinical [51]
Probiotic intervention associated with sleep quality and stress-related biomarkers	Clinical interventional study	Individuals with sleep or stress-related complaints	Improved sleep quality and modulation of stress-related biomarkers	Human clinical [52]
Probiotic intervention in sleep-deprivation-related anxiety/depression model	Preclinical/experimental study	Animal model of sleep deprivation	Attenuation of anxiety- and depression-like behaviors through inflammatory and circadian-related pathways	Preclinical [53]
Probiotic intervention modulating tryptophan–serotonin pathways	Clinical or translational study	Mood, stress, or cognitive-performance context	Improvement in mood and cognitive performance through modulation of tryptophan–serotonin metabolism	Human/translational evidence [54]
Clock-gene-targeted probiotic strategy with melatonin-like effects	Preclinical study	Experimental animal model	Melatonin-like effects on circadian rhythmicity and cognitive performance	Preclinical [55]

**Table 3:** Metabolomic alterations potentially relevant to biological stratification in major depressive disorder.

Metabolite Class	Representative Metabolites	Alteration Reported in MDD or Mood-Disorder Studies	Biological Interpretation	Potential Stratification Relevance
Tryptophan–kynurenine pathway	Tryptophan, kynurenine, kynurenic acid, quinolinic acid	Reduced tryptophan availability and/or shift toward kynurenine pathway in inflammatory depressive phenotypes	Links immune activation, serotonin/melatonin availability, glutamatergic signaling, and neurotoxicity	May identify an inflammation-related depressive subtype
Amino acids and neurotransmitter-related metabolites	Glutamate, GABA, branched-chain amino acids, aromatic amino acids	Altered excitatory/inhibitory balance and amino acid metabolism	Reflects neurotransmission, stress physiology, energy balance, and microbial amino acid metabolism	May help distinguish symptom dimensions such as anxiety, anergia, cognitive impairment, or affective instability
Lipids and glycerophospholipids	Phosphatidylcholines, sphingolipids, fatty-acid derivatives	Altered lipid membrane and inflammatory signaling profiles	Suggests membrane remodeling, oxidative stress, and inflammatory dysregulation	May support biological subtyping and prediction of treatment response
Energy metabolism	Citrate, lactate, pyruvate, ketone bodies, TCA-cycle intermediates	Perturbations in mitochondrial and bioenergetic pathways	Supports the role of impaired cellular energy metabolism in depressive symptoms	May identify fatigue/anergia-dominant or metabolically vulnerable phenotypes
Short-chain fatty acids	Acetate, propionate, butyrate	Altered production or signaling in dysbiosis-related states	Reflects microbial fermentation, intestinal barrier integrity, immune regulation, and peripheral clock signaling	May index microbiota-dependent chrono-metabolic status
Bile acids	Primary and secondary bile acids	Altered bile acid pools, microbial conversion, or rhythmicity	Links hepatic clocks, microbial metabolism, enterohepatic circulation, and metabolic inflammation	May identify metabolic/circadian subtypes
One-carbon and methylation-related metabolites	Betaine, choline, methionine, folate-related metabolites	Altered methylation and redox-related metabolic profiles	Relevant to epigenetic regulation, including potential clock-gene methylation	May support epigenetic–metabolic stratification
Oxidative stress and redox metabolites	Glutathione-related metabolites, 2-hydroxybutyrate, oxidative lipid derivatives	Evidence of oxidative stress and impaired redox balance	Reflects inflammatory burden, mitochondrial dysfunction, and cellular stress	May identify high-inflammatory or high-oxidative-stress subgroups

Abbreviations: MDD, major depressive disorder; TCA, tricarboxylic acid.

**Table 4:** Summary of clinical and preclinical evidence on circadian-microbiota-metabolite-mood interactions.

Mechanistic Domain	Study Design	Population/Model	Intervention/Exposure	Main Findings Related to Circadian–Microbiota–Mood Interactions	Evidence Level
Gut microbiota rhythmicity	Human observational and preclinical studies	Healthy humans and animal models	Time-of-day sampling; feeding–fasting cycles; circadian alignment or disruption	Gut microbial abundance and metabolic function show daily oscillations. Feeding–fasting cycles act as major entraining signals, whereas circadian disruption may blunt microbial rhythmicity and alter host metabolic homeostasis [22,23,24].	Human and preclinical
Diet, microbiota, and sleep	Human observational and dietary studies	General population and sleep-related cohorts	Mediterranean diet, fiber-rich diet, fruit and vegetable intake, prebiotic/polyphenol exposure	Dietary patterns rich in fibers, prebiotics, and polyphenols may support SCFA-producing taxa and are associated with better sleep quality and shorter sleep-onset latency [26,27].	Human clinical/observational
Sleep disruption and microbiota composition	Preclinical experimental studies	Animal models	Sleep fragmentation or chronic sleep disruption	Sleep fragmentation can alter gut microbial composition, including changes in the Firmicutes/Bacteroidetes ratio, supporting bidirectional communication between sleep physiology and microbiota structure [28].	Preclinical
SCFAs and peripheral clock regulation	Preclinical studies and limited human supplementation data	Animal models; human supplementation studies	SCFA production or supplementation, including butyrate	SCFAs may modulate peripheral clock gene expression, act through *FFAR2*/*3* signaling, influence HDAC activity, support blood–brain barrier integrity, and affect synaptic plasticity. Butyrate supplementation has been associated with changes in core clock gene expression [29,30,31,32,33,34].	Mainly preclinical; limited human evidence
Tryptophan–kynurenine pathway	Clinical and translational studies	Patients with depressive symptoms or inflammatory phenotypes; experimental models	Stress, inflammation, altered tryptophan availability, diet or fasting-related modulation	Inflammation may divert tryptophan from serotonin/melatonin synthesis toward the kynurenine pathway, increasing neuroactive metabolites such as quinolinic acid and contributing to sleep disturbance, neuroinflammation, and depressive symptoms [35,36].	Human/translational
Bile acids and circadian metabolism	Human chronobiological studies and mechanistic studies	Healthy humans; experimental models	Meal timing, light–dark cues, bile acid rhythmicity, hepatic clock regulation	Circulating bile acids show rhythmic profiles influenced by meals and environmental cues. Hepatic bile acid synthesis and enterohepatic circulation interact with clock-related regulators such as Rev-ERBα and RORα, linking circadian biology, microbial metabolism, and metabolic risk [37,38,39].	Human and mechanistic
Microbial-derived neuroactive compounds	Translational and mechanistic studies	Human gut–brain axis literature; experimental models	Microbial production or modulation of GABA, serotonin, dopamine, and related pathways	Microbial-derived or microbiota-modulated neuroactive compounds may influence vagal signaling, neurotransmitter availability, and synaptic plasticity. These mechanisms are biologically plausible but remain incompletely validated in mood-disorder populations [40].	Translational/mechanistic
HPA axis, intestinal barrier, and inflammation	Clinical and preclinical studies	Mood-disorder populations; animal models of stress or dysbiosis	Dysbiosis, chronic stress, leaky gut, LPS translocation, cytokine activation	Dysbiosis and circadian disruption may contribute to HPA axis hyperactivity, flattened cortisol rhythmicity, intestinal barrier impairment, systemic inflammation, and neuroinflammatory signaling relevant to mood disorders [3,24,30,31].	Human and preclinical
BDNF, synaptic plasticity, and neuroinflammation	Mainly preclinical studies	Animal and cellular models	Butyrate, acetate, secondary bile acids, inflammatory or dysbiotic conditions	Butyrate and acetate may influence histone acetylation, neuroinflammation, *BDNF* expression, and synaptic plasticity. These findings provide mechanistic support but should not be interpreted as direct clinical proof in mood disorders [41,42].	Mainly preclinical
Clock genes, neuropeptides, and monoaminergic metabolism	Preclinical and mechanistic studies	Animal models with clock-gene alterations	CLOCK mutations; altered CCK, orexin, ghrelin, *MAOA* regulation	Clock-gene disruption may alter neuropeptide rhythmicity and monoaminergic metabolism. These pathways may contribute to affective instability, although clinical validation remains limited [40,43].	Preclinical/ mechanistic
Mitochondrial and redox pathways	Translational and mechanistic studies	Psychiatric populations and experimental models	Circadian redox coupling; mitochondrial dysfunction	Circadian regulation is linked to cellular redox status and mitochondrial function, suggesting a possible connection between chrono-disruption, bioenergetic impairment, and psychiatric vulnerability [44,45].	Translational/ mechanistic
Chrononutrition and time-restricted feeding	Mainly preclinical interventional studies	Obese mice, Drosophila, early-life animal models	Early or late time-restricted feeding; high-fat diet exposure	Time-restricted feeding may improve metabolic and circadian parameters in some animal models, but effects may depend on timing, developmental period, and metabolic context. Psychiatric applicability remains to be established [46,47,48,49].	Mainly preclinical
Chronobiotics and probiotics	Human randomized trials and preclinical studies	Patients with MDD; individuals with sleep/stress symptoms; animal models	Multispecies probiotics; psychobiotic or chronobiotic interventions	Probiotic interventions may modulate depressive symptoms, sleep quality, fatigue, stress biomarkers, inflammatory pathways, tryptophan–serotonin metabolism, and possibly clock gene expression. Human evidence remains limited and often formulation-level rather than strain-specific [50,51,52,53,54,55].	Human and preclinical
Psychotropic drugs and microbiota	Clinical observational and translational studies	Patients receiving antipsychotics or antidepressants	Atypical antipsychotics, SSRIs, pharmacological exposure	Psychotropic medications may be associated with specific microbial signatures, altered alpha diversity, and microbial/metabolomic profiles linked to treatment response, supporting the potential of microbiota-informed precision psychiatry [56,57,58].	Human clinical/translational
Metabolomics and treatment stratification	Clinical metabolomic and multi-omic studies	Patients with MDD or mood disorders	Metabolomic profiling; microbial and metabolic biomarker assessment	Metabolomic profiles may stratify MDD into biologically distinct subtypes and may contribute to prediction of treatment response, although standardization and validation in larger cohorts are required [62,63,64,65,66,67].	Human clinical/translational

Abbreviations: *BDNF*, brain-derived neurotrophic factor; CCK, cholecystokinin; *FFAR2*/*3*, free fatty acid receptors 2 and 3; GABA, gamma-aminobutyric acid; HDAC, histone deacetylase; HPA, hypothalamic–pituitary–adrenal; LPS, lipopolysaccharide; *MAOA*, monoamine oxidase A; MDD, major depressive disorder; RORα, retinoic acid receptor-related orphan receptor alpha; Rev-ERBα, nuclear receptor subfamily 1 group D member 1 (*NR1D1*); SCFAs, short-chain fatty acids; SSRIs, selective serotonin reuptake inhibitors.

## Data Availability

No new data were created.

## Abbreviations

The following abbreviations are used in this manuscript:

GABA	gamma-aminobutyric acid
MD	mood disorder
SCN	suprachiasmatic nucleus
VN	vagus nerve
MDD	Major Depressive Disorder
BD	Bipolar Disorder
CLOCK	Circadian Locomotor Output Cycles Kaput
NPAS2	neuronal PAS 2
BMAL1	Brain and Muscle Arnt-like Protein 1
Per	Period
Cry	Cryptochrome
NAc	nucleus accumbens
SCFAs	Short-Chain Fatty Acids
FFAR 2/3	free fatty acid receptors 2/3
PYY	peptide YY
GLP-1	glucagon-like peptide-1
BBB	Blood–Brain Barrier
CREB	cAMP Response Element-Binding protein
HDAC	Histone Deacetylase
IDO	enzyme indoleamine 2,3-dioxygenase
BSH	Bile Salt Hydrolase
DA	Dopamine
SERT	Serotonin Transporter
MAO	Monoamine Oxidase
LPS	Lipopolysaccharides
NT	neurotransmitter
BDNF	Brain-Derived Neurotrophic Factor
HPA	Hypothalamic–Pituitary–Adrenal
TRF	time-restricted feeding
